# Isolation, Antibiogram and Factors Associated With *Staphylococcus aureus* From Fish at the Landing Site and Selected Restaurants in Central Gondar, Ethiopia: One Health Approach

**DOI:** 10.1002/vms3.70880

**Published:** 2026-03-13

**Authors:** Misganaw Tezera Ayele, Asnakew Mulaw Berihun, Ambaye Kenubih Worku, Mengesha Ayehu Getnet

**Affiliations:** ^1^ Department of Pathobiology College of Veterinary Medicine and Animal Sciences, University of Gondar Gondar Ethiopia

**Keywords:** antimicrobial, fish, isolation, Lake Tana, restaurants, *Staphylococcus aureus*

## Abstract

**Background:**

In developing countries, foodborne infections, including those caused by *Staphylococcus aureus*, pose a significant public health risk.

**Objective:**

This study aimed to isolate *S. aureus*, assess its antimicrobial resistance and identify factors influencing its prevalence in unloading sites and restaurants in central Gondar, Ethiopia.

**Method:**

A cross‐sectional study (December 2023–September 2024) was conducted with a total of 301 fish and swab samples were purposively collected. Samples were analysed for *S. aureus* using culture, Gram staining, biochemical tests and Kirby–Bauer antimicrobial susceptibility testing. Data were analysed using STATA 16 with descriptive statistics at *p* < 0.05.

**Result:**

From a total of 301 samples were collected from fish, hands and knives at unloading sites, with *S. aureus* found in 26% of the samples. Of the 201 fish samples, 13% of unfilleted and 39% of filleted fish tested positive. A questionnaire survey revealed that all respondents handled fish with bare hands. Antibiotic testing showed that *S. aureus* was susceptible to ciprofloxacin (100%) and chloramphenicol (92%) but highly resistant to erythromycin (55%), tetracycline (72%) and penicillin G (87%). Additionally, 57% of isolates were multidrug‐resistant.

**Conclusion:**

Over one‐fourth of samples were contaminated with *S. aureus*, mainly due to poor hygiene and bare‐hand handling, with high resistance to penicillin and tetracycline observed. Strengthened hygiene practices, routine resistance monitoring and continuous food safety training are recommended.

## Graphical abstract

This study identified *Staphylococcus aureus* in 26% of fish, hand and knife samples from Gondar, Ethiopia, with high multidrug resistance. Findings highlight unsafe handling practices and recommend improved hygiene, regular monitoring and targeted antibiotic use to reduce contamination risks and protect public health through a One Health approach.

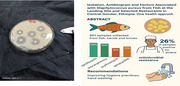

## 1 Introduction

### 1.1 Background

Fish plays a vital role in the human diet, with an ever‐growing global demand. Over the past 60 years, world fish production has dramatically increased, reaching approximately 179 million tons in 2018, with a value of $401 billion. Similarly, global fish consumption rose from 9.0 kg per capita in 1961 to 20.5 kg in 2018, marking a significant transformation in the fishery industry (Hussein 2014; FAO 2018). In Africa, fish contributes 19% of animal protein and essential micronutrients, particularly fatty acids that cannot be replaced by other food commodities (Quinlan 2013). Fish consumption in Africa averages 10.8 kg per person per year, whereas in Ethiopia it is significantly lower at just 0.2 kg per person per year (Breuil and Grima 2014).

Ethiopia, being a landlocked nation, relies entirely on its inland lakes, reservoirs and rivers for fishing resources (Seo et al. 2007). The country's annual fish production potential is estimated at 51,400 t (Mainous et al. 2006). However, the domestic fish market is relatively small outside major fishing regions. Most lakes are located within the East African Rift Valley system, with Lake Tana being the largest, accounting for over half of Ethiopia's inland water area (Alazar 2016).

Fish is an ideal dietary option due to its high nutritional value and easy digestibility (Adugna et al. 2019). However, fish meat is highly susceptible to various bacterial infections, many of which are pathogenic, whereas others are saprophytic in nature (Bujjamma and Padmavathi 2015). Bacterial pathogens in fish include zoonotic and pathogenic bacteria such as *Edwardsiella*, *Salmonella*, *Escherichia coli*, *Staphylococcus aureus*, *Vibrio* and *Aeromonas*. These pathogens have been isolated from fish in various parts of Ethiopia (Otte and Pica‐Ciamarra 2021). Fish diseases caused by bacterial infections include dropsy, epizootic ulcerative syndrome (EUS), swim bladder disease, scale loss disease, fin rot and tail disease (Adugna et al. 2019). Most pathogenic bacteria are naturally occurring saprophytes and opportunistic pathogens that invade fish tissue under favourable conditions (Hussein 2014).

Zoonotic diseases are estimated to cause 2.5 billion cases of human illness globally every year (Salyer et al. 2017). More than 60% of existing and 75% of emerging or re‐emerging human diseases are zoonotic, with 36% of these diseases linked to food‐producing animals (Otte and Pica‐Ciamarra 2021).

*S. aureus* is a major cause of foodborne illnesses, primarily through the consumption of preformed staphylococcal enterotoxins. These enterotoxins are highly heat‐stable and often associated with staphylococcal foodborne intoxication (SFI) (Dabassa et al. 2019). Prepared foods containing more than 10^3^ colony‐forming units per gram (cfu/g) of *S. aureus* are considered unsatisfactory, and counts exceeding 10^4^ cfu/g render the food potentially harmful for consumption. Consuming food contaminated with staphylococcal enterotoxins in amounts as small as nanograms to micrograms can cause severe illness, ranging from mild skin infections to life‐threatening conditions (Seo et al. 2007).

Improper refrigeration or exposure to elevated temperatures during food processing often creates conditions favourable for the growth of *S. aureus* (Dabassa et al. 2019). The bacterium colonizes 30%–50% of the healthy human population, with the anterior nares of the nose being the most common carriage site (Wertheim et al. 2005). According to the National Health and Nutrition Examination Survey (2001–2002) in the United States, approximately 32.4% of the non‐institutionalized population, including children and adults, were nasal carriers of *S. aureus* (Mainous et al. 2006).

Preventing staphylococcal food poisoning can be challenging, as carriers often exhibit no symptoms. A cross‐sectional study conducted in Gondar revealed that 16.5% of fingernail samples from 127 food workers in cafeterias tested positive for *S. aureus* (Andargie et al. 2008). Similarly, in Botswana, 57.5% of 200 food workers tested positive for *S. aureus* (Loeto et al. 2007).

Globally, antimicrobial‐resistant *S. aureus* poses a significant threat to public health. Unhygienic and improper food processing practices are major contributing factors to the emergence of resistant strains (Quinlan 2013). In developing countries like Ethiopia, where raw fish consumption is common, antimicrobial‐resistant *S. aureus* strains are an emerging concern (Dabassa et al. 2019). The objective of the current research is to provide baseline data on the status of *S. aureus* along the fish value chain in Gondar town and contribute, if possible, to the development of national food safety strategies (Mulder et al. 2020).

### 1.2 Statement of the Problem and Justification

Contaminated food poses a significant risk to public health, causing billions of illnesses and thousands of deaths annually (Quinlan 2013). Cross‐contamination during food preparation in food service establishments is a leading cause of foodborne diseases (FBD). Fish, being a common source of FBD, is particularly concerning in developing nations due to poor hygiene practices and lack of awareness. In Ethiopia, inadequate food safety regulations, weak institutions and insufficient training for food handlers contribute to the high incidence of foodborne illnesses, particularly from animal and fish products (Dabassa et al. 2019). Fish, being highly perishable, requires careful handling to ensure safety and quality. In Lake Tana, fish processing is done using traditional methods in unsanitary conditions, emphasizing the need for research on bacterial contaminants like *S. aureus*. This study aims to assess hygienic practices, antibiotic resistance and public health risks associated with fish products in Gondar city, providing critical data for developing strategies to mitigate fish‐related FBD.

### 1.3 Objective of the Study

#### 1.3.1 General Objective

- To isolate *S. aureus* and assess its antibiogram and factors associated for its occurrence in unloading sites and selected restaurants of Gondar city North west Ethiopia.

#### 1.3.2 Specific Objectives

- To isolates *S. aureus* from fresh and ready to eat fish at unloading sites and restaurants, respectively.
- To assess the antimicrobial susceptibility pattern of *S. aureus*.
- To assesses factors associated with fishing activity and food safety in study sites.

## 2 Materials and Methods

### 2.1 Study Area

**FIGURE 1:**
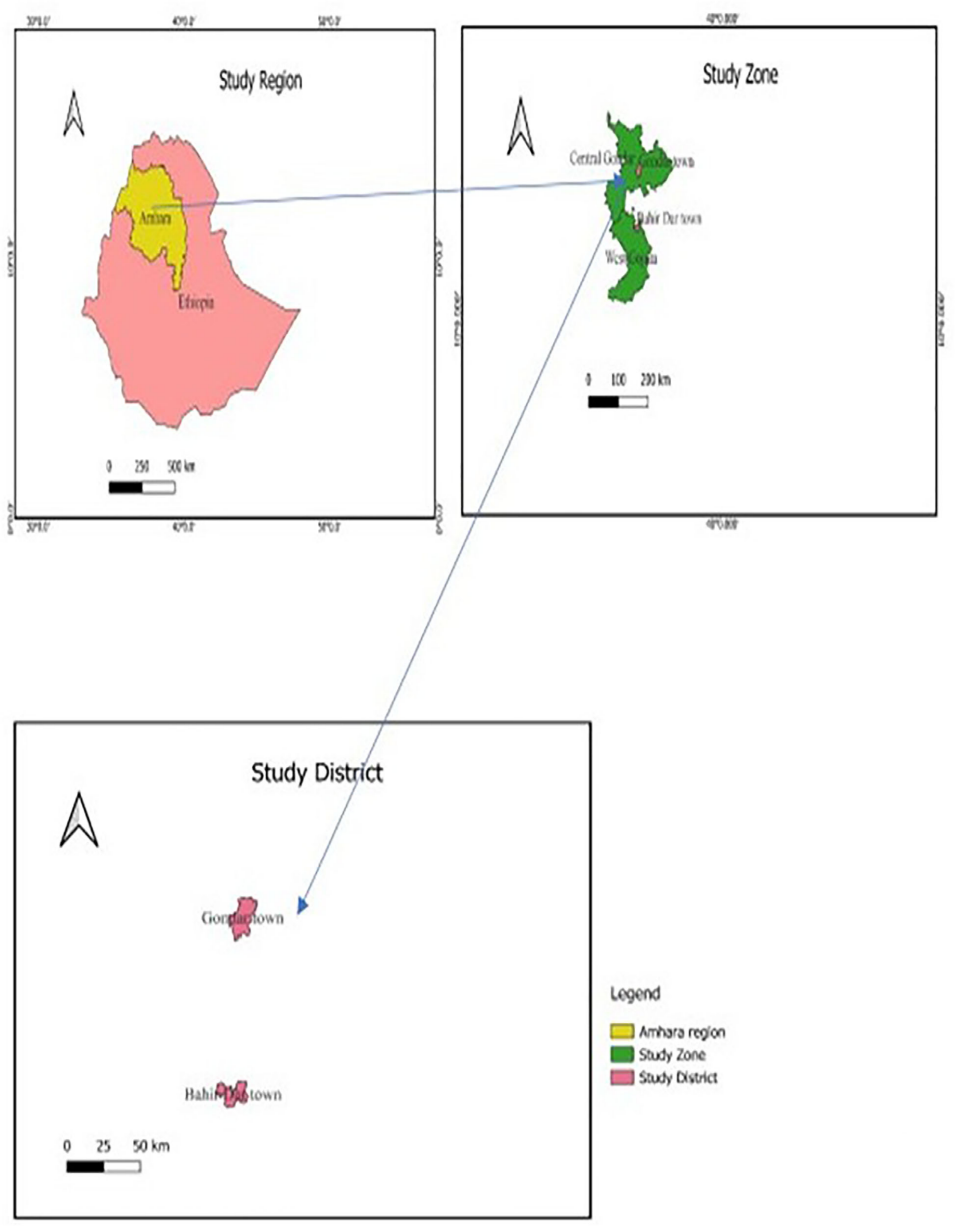
A map showing the study area. *Source*: GIS software (2020).

As shown in Figure 1, the study was conducted in Gondar city (restaurants) and in Lake Tana unloading sites (Miterha, Sheha and Gorgora). The city of Gondar is situated in north‐western parts of Ethiopia, Amhara Regional State. It is at 12°3″ N latitude and 37°28″ E longitude. Gondar is located at 727 km from Addis Ababa, the capital city of federal government of Ethiopia, and 120 km from Bahir Dar, the capital city of Amhara National Regional State. Gondar has five sub cities and a total area of 192.3 km^2^ with undulating mountainous topography. According to the 2023 National Population and Housing Census estimation Gondar consists of a total of 675,651 peoples. Gondar is the centre of political and economic activities of the North Amhara region and it is main city of the central Gondar Zone. It stands at an elevation of 7500 ft (2300 m) on a basaltic ridge from which streams flanking the town flow to Lake Tana, 21 miles (34 km) Gondar City Administration (2015).

Lake Tana is the head quarter water source of Blue Nile River and is the largest fishing sites in the region and the country which is almost dominated by artisanal fishermen. This lake is found in Amhara Region and has a surface area of 32,000 km^2^ with a maximum and minimum depth of 14 and 8 m, respectively. The Lake provides commercially important fish groups; namely, African catfish (*Clarias gariepinus*) locally called ‘Ambaza’, Nile tilapia (*Oreochromis niloticus*) locally called ‘Kereso’ and *Labeobarbus* spp. (Cyprinidae) locally called ‘Nech Asa’. The study was carried out at Gondar town and fish landing site around Gondar As shown in Figure 1, which was obtained from geographical information system software 2020 shape file sketch.

### 2.2 Study Population and Sample Type

The study population consists of raw fish (both fresh unfilleted and filleted), including species such as Nile tilapia (*O. niloticus*), *Labeobarbus* (Cyprinidae) and catfish (*C. gariepinus*), which were collected by fishermen from Lake Tana. Additionally, frozen and cooked fish (ready‐to‐consume) were included, such as **Fried fish**: A dish prepared by gutting or removing inedible parts of the fish, followed by thorough cooking in oil at high temperatures for human consumption. **Undercooked fish**: A dish made by cutting the muscle into pieces, adding spices and cooking at low temperatures. **Fish stew**: A dish prepared by mincing the fish muscle, adding spices and cooking at high temperatures for human consumption. The types of samples included in the study were as follows: fish meat swabs from raw and prepared fish, hand swabs from fisheries and restaurant workers and knife swabs from knives used for fish processing.

### 2.3 Study Design and Sample Size Determination

A cross‐sectional study was conducted from December 2023 to September 2024. In addition, an observational checklist and a pre‐tested questionnaire were administered to workers along the value chain, including restaurants, to determine possible sources and sites of contamination within the value chain. The sample size was determined using the Thrusfield formula (Thrusfield 2005), with a calculated total sample size of 384. However, as the fish population in Lake Tana was unknown, the sample distribution was based on data from the Gondar Zuria Wereda Fishery Sector. According to this data, fisheries collect 10% *catfish*, 30% *Labeobarbus* and 60% *Nile tilapia*. Therefore, a total of 301 samples were investigated.

### 2.4 Sampling Method

Twelve restaurants were selected using purposive sampling from five sub‐cities. These five sub‐cities, identified based on Gondar City Administration data (2015), include Arada Kefle Ketema, Azezo Teda Kefle Ketema, Zobel Kefle Ketema, Facile and Maracie Kefle Ketema. These restaurants were chosen based on their popularity in fish meals/food, customer flow, accessibility and willingness to participate in the research.

In Lake Tana, three landing sites Mitreha, Sheha and Gorgora located in Gondar Zuria Wereda and West Denbia Wereda were also selected through purposive sampling. These sites were identified as key locations due to their high levels of fishing activity, with fish being landed regularly twice per day (morning and afternoon), ensuring consistent availability of samples. They also offer a broader spatial understanding of fishing practices and fish handling. Accessibility and logistical feasibility were additional factors in their selection, as these sites are relatively easier to reach and have basic infrastructure that supports sample collection. Furthermore, the presence of established and cooperative fishing communities at these sites made it feasible to engage with fishers and obtain reliable data.

A total of 301 samples were collected, including 201 samples from unloading sites and 100 samples from restaurants. These were gathered over a total of 18 observations: 12 observations for collecting swab samples from unloading sites and 6 observations for collecting swab samples from restaurants in Gondar City.

### 2.5 Data Collection Procedure

#### 2.5.1 Sample Collection

Hand swab and knife swab samples were collected from selected restaurants using sterile sampling bottles containing buffered peptone water and kept in an icebox with ice packs. Similarly, swab samples from raw or fresh fish at selected fishing sites were collected and placed in sterile sampling bottles containing buffered peptone water. Sterile cotton swabs were used to transfer the fish swab from the uncooked fish meat plate to the sampling containers.

After labelling and coding with all necessary information, the samples were immediately transported to the University of Gondar College of Veterinary Medicine and Animal Sciences, Veterinary Microbiology Laboratory, using an icebox with ice packs. The samples were processed within 24 h of collection, and all inoculums were incubated overnight at 37°C for the isolation and identification of *S. aureus* from fish meat swabs and contact surface sampling swabs.

#### 2.5.2 Questionnaire Survey and Observation

A pre‐tested questionnaire was designed to collect separate data (Supporting Information Annexes V and VI) for fish processors in restaurants and fish harvesters at unloading sites. Questionnaire surveys were conducted to assess factors associated with cross‐contamination in raw and ready‐to‐eat fish‐derived foods. The questionnaire also included socio‐demographic factors, transport‐related issues, educational status and personal hygiene practices.

Respondents were purposively selected based on their significant roles in food processing and handling. The questionnaire and observational checklists were administered in accordance with the standard guidelines of the Codex Alimentarius Commission of the Food and Agriculture Organization (Supporting Information Annex VII) (FAO 2009).

As provided in the supportive files in Supporting Information Annexes V and VI, the structured questionnaires were designed to fish processors in hotels and restaurants and fish harvesters at fish unloading sites.

For hotel and restaurant workers, example questions included ‘Do you wash your hands before and after handling fish?’, ‘Do you clean contact surfaces before starting food preparation?’, ‘Do you wear gloves or aprons during food handling?’ and ‘Have you received food hygiene training?’ For fish harvesters at unloading sites, questions such as ‘Do you clean your fishing equipment before and after use?’ were included to assess food handling practices at the primary stage of the supply chain.

To quantify practices and knowledge levels, a binary scoring system was applied. Each response was recorded as either ‘Yes’ or ‘No’, where a ‘Yes’ answer indicating a safe or desirable practice was awarded one (1) point, and a ‘No’ answer indicating an unsafe or undesirable practice received zero (0) points. A total score was then calculated for each respondent. Higher scores reflected better awareness and safer practices, whereas lower scores indicated inadequate food safety knowledge or poor handling behaviours.

### 2.6 Isolation and Biochemical Tests

The assignment of *S. aureus* species and final identification of staphylococcal organisms were performed using various culturing methods, followed by Gram staining and biochemical tests. The biochemical tests included the catalase test, tube coagulase test, slide coagulase test, Voges–Proskauer (VP) test and mannitol sugar fermentation test. Both coagulase tests, using rabbit plasma, were conducted in parallel to further confirm the identification of *S. aureus*.

#### 2.6.1 Gram's Staining

Gram staining was performed on all suspected *Staphylococcus* species cultures, and the sizes, shapes and cell configurations of the cultures were examined under a light microscope. Presumptive *Staphylococcus* species were identified based on Gram‐stained smears of typical colonies, which revealed Gram‐positive cocci arranged in irregular grape‐like clusters.

#### 2.6.2 Catalase Test

Using a bacteriological loop, pure cultures of the isolates to be tested for catalase were removed from the agar plate and mixed with a drop of 3% hydrogen peroxide on a sanitized slide. Within a few seconds, bubbles of oxygen were released, indicating a positive reaction and the presence of *S. aureus* (Quinn et al. 2002).

#### 2.6.3 Mannitol Salt Agar (Mannitol Fermentation)

The colonies were streaked onto Mannitol Salt Agar plates and incubated at 37°C. Growth was checked after 24–48 h. These colonies were confirmed through Gram staining, haemolysis on blood agar, colony characterization and a positive catalase test. The presence of growth and a pH shift from red to yellow in the medium indicated the presence of coagulase‐positive *S. aureus*. Fermentation of mannitol by *S. aureus* causes the medium to turn yellow within 24 h of incubation (Quinn et al. 2002).

#### 2.6.4 Coagulase Test

Both slide coagulase and tube coagulase tests were used as coagulase assays. *S. aureus* presumed to be identified from Mannitol Salt Agar was subcultured onto a nutrient agar plate. After 24 h, the culture colonies of *S. aureus* were selected using a bacteriological loop, placed on a clean slide and emulsified. A drop of rabbit plasma was added to the test suspension, and it was thoroughly mixed with a wire loop for 5–10 s. Clumping of the cocci was interpreted as a positive result (Quinn et al. 2002). For the tube coagulase test, 0.5 mL of selected *Staphylococcus* isolates cultured in tryptic soy broth at 37°C for 24 h was added to 0.5 mL of rabbit plasma in sterile tubes. This test was conducted for those isolates that were negative in the slide coagulase test. Any visible clotting inside the tube, ranging from a loose to a firm clot that remained immovable when the tube was inverted (tilted), was considered a positive result. No clotting at all was interpreted as a negative result (Quinn et al. 2002).

#### 2.6.5 VP Test

The VP test is a biochemical test that detects the ability of bacteria to metabolize pyruvate into a neutral intermediate product called acetylmethylcarbinol or acetoin. The test is performed by adding alpha‐naphthol and potassium hydroxide to the VP broth. This test is conducted on Gram‐positive, catalase‐positive species to identify coagulase‐positive *S. aureus* (Quinn et al. 2002).

### 2.7 Antimicrobial Susceptibility Profile

All isolates of *S. aureus* were subjected to an antibiotic susceptibility test using the Kirby–Bauer agar disc diffusion method, following the Clinical Laboratory Standards Institute (CLSI) guidelines of the USA, on Mueller–Hinton agar (MHA). The antibiotics were selected based on their availability and relevance for routine testing and reporting on non‐fastidious organisms. One representative antibiotic from each subclass of commonly used and widely available antibiotics for treating staphylococcal‐related diseases in both animals and humans was chosen. On the basis of these criteria, seven antibiotics were selected for this study: chloramphenicol (30 µg), ciprofloxacin (5 µg), vancomycin (30 µg), erythromycin (15 µg), gentamicin (10 µg), tetracycline (30 µg) and penicillin (10 U) (CLSI 2020).

For the susceptibility test, three to five well‐isolated colonies of the same morphological type were selected from a nutrient agar plate culture and transferred into test tubes containing sterile saline. The suspension was mixed thoroughly, and the density was adjusted to 0.5 McFarland using saline or additional *S. aureus* colonies. A sterile swab was dipped into the suspension, and the excess inoculum was removed by pressing it against the sides of the tube to prevent over‐inoculation of the plates. The inoculum was spread evenly over the entire surface of the agar plate by swabbing in three directions. Antibiotic discs were applied firmly onto the agar surface, and the plates were incubated for 24 h at 37°C. The diameter of the zone of inhibition around each disc was measured using a ruler in millimetres (mm) and interpreted according to the CLSI standards as susceptible, intermediate or resistant. Isolates showing resistance to three or more antibiotics were considered multiple drug‐resistant (MDR) (Beyene et al. 2022).

### 2.8 Data Analysis

All data collected during the study period were checked, coded and entered into an Excel spreadsheet before being analysed using STATA software version 16 (Texas, USA). Descriptive statistics, such as percentages and proportions, were used to compute the number of fish samples positive for *S. aureus*. A univariable logistic regression model was employed, and variables with a *p* value of <0.05 were exported to a multivariable logistic regression model to assess the effects of potential confounders. The degree of association between risk factors and the occurrence of *S. aureus* in fish samples was quantified using the adjusted odds ratio obtained from the multivariable logistic regression models. In all analyses, the confidence level was set at 95%, and a *p* value of less than 5% (*p* < 0.05) was considered statistically significant.

## 3 Results

### 3.1 Occurrence of *Staphylococcus aureus*

The current study was observed the occurrence of *S. aureus* and, out of 301 samples analysed, 79 (26%) tested positive for *S. aureus*, with 53 (26%) from fish and 26 (26%) from hand and knife swabs. Although the contamination rates appear equal, a statistically significant difference was observed (OR = 2.2, *p* = 0.024, 95% CI: 1.103–1.474) (Table 1). This suggests that fish samples had more than twice the odds of contamination compared to contact surfaces. The result indicates fish may serve as a primary source of *S. aureus* contamination. This highlights the need for improved hygiene during fish handling and storage.

**TABLE 1:** Occurrence of *Staphylococcus aureus* from fish and contact surfaces.

Sampling site	No. of samples (*N* = 301)	No. of positives (%) (*n* = 79)	OR	*p* value	95% CI
On fish	201	53 (26%)			
On hand and knife	100	26 (26%)	2.2	0.024	1.10304–1.4739
**Total**	301	79 (26%)			

*Note*: Statistically significant at *p* < 0.05.

Abbreviations: CI = confidence interval; OR = odds ratio.

### 3.2 Isolation of *Staphylococcus aureus* Related to Species

From 201 fish samples examined, *S. aureus* was isolated from three different fish species with varying rates: 36% (10/28) in African catfish, 27% (13/48) in *Labeobarbus* species and 24% (30/125) in Nile tilapia. Although the highest contamination rate was observed in African catfish, the difference in *S. aureus* occurrence among the three species was not statistically significant (OR = 1.28, *p* = 0.272, 95% CI: 0.823–1.996) (Table 2). This suggests that *S. aureus* contamination is not strongly associated with a specific fish species in this study. The results indicate that all fish types are similarly at risk for contamination, and food safety interventions should not be species‐specific but rather applied broadly across all fish handling and processing activities.

**TABLE 2:** Isolation of *Staphylococcus aureus* related to species.

Fish species	No. of samples (*n* = 201)	No. of positive (%) (*n* = 53)	OR	*p* value	95% CI
Catfish	28	10 (36%)	1.281561	0.272	0.823–1.996
*Labeobarbus*	48	13 (27%)			
Nile tilapia	125	30 (24%)			
Total	201	53 (26%)			

*Note*: Statistically significant at *p* < 0.05.

Abbreviations: CI = confidence interval; OR = odds ratio.

### 3.3 Isolation of *Staphylococcus aureus* From Fish Samples at Different Sampling Sites

As shown in Table 3, the results show variation in the occurrence of *S. aureus* across different sample types and locations. Among raw fish samples, 13% (13/97) of unfilleted and 39% (40/104) of filleted fish were positive for *S. aureus*. This difference was statistically significant (OR = 2.98, *p* = 0.026, 95% CI: 1.139–7.797), indicating that filleted fish had nearly three times higher odds of contamination than unfilleted fish. Across the three fish unloading sites, *S. aureus* was detected in 36% of samples from Miterha, 24% from Gorgora and 19% from Sheha. However, the differences between these sites were not statistically significant (OR = 1.24, *p* = 0.157, 95% CI: 0.921–1.663). Notably, when comparing contamination at unloading sites versus restaurants, a significant difference was observed. Unloading sites had a 26% contamination rate (53/201), whereas restaurant samples showed the same proportion (26%), yet with a significant odds ratio (OR = 2.2, *p* = 0.024, 95% CI: 1.109–4.366), suggesting a higher likelihood of contamination linked to handling and processing environments.

**TABLE 3:** Isolation of *Staphylococcus aureus* from samples at different sampling sites.

Sample type and sample site	No. of samples (*n* = 301)	No. of positives (%) (*n* = 79)	OR	*p* value	95% CI
Raw unfilleted	97	13 (13%)			
Raw filleted	104	40 (39%)	2.980519	0.026	1.139–7.797
Total	201	53 (26%)			
Miterha	67	24 (36%)			
Sheha	67	13 (19%)	1.237537	0.157	0.921–1.663
Gorgora	67	16 (24%)			
Gondar	100	26 (26%)			
Total	301	79 (26%)			
In unloading sites	201	53 (26%)	2.2	0.024	1.109–4.366
In restaurants	100	26 (26%)			
Total	301	79 (26%)			

*Note*: Statistically significant at *p* < 0.05.

Abbreviations: CI = confidence interval; OR = odds ratio.

### 3.4 The Occurrence of *Staphylococcus aureus* From Different Swab Sites

The results in Table 4 indicate the occurrence of *S. aureus* contamination across different swab sites. Among the 301 total samples, *S. aureus* was detected in 20% (10/50) of hand swabs, 32% (16/50) of knife swabs and 26% (53/201) of fish meat swabs. Although knife swabs showed the highest contamination rate, the difference in contamination among the three swab sites was not statistically significant (OR = 0.98, *p* = 0.921, 95% CI: 0.652–1.472). This suggests that contamination may occur at multiple points during handling and processing, but no specific surface or tool showed significantly higher risk.

**TABLE 4:** The occurrence of *Staphylococcus aureus* from different swab site.

Swab site	No. of samples (*n*)	No. of positives (%)	OR	*p* value	95% CI
Hand swab	50	10 (20)			
Knife swab	50	16 (32)	0.98	0.921	0.652–1.472
Fish meat swab	201	53 (26)			
**Total**	**301**	**79 (26)**			

*Note*: Statistically significant at *p* < 0.05.

Abbreviations: CI = confidence interval; OR = odds ratio.

### 3.5 Isolation and Identification of *Staphylococcus aureus* Species

The present study found, out of the 301 total fish, hand and knife swab samples, a progressive identification process was followed using standard biochemical tests. Beta‐haemolysis was observed in 40% of the samples, serving as a preliminary indicator of potential *Staphylococcus* species. Among these, 102 (85%) showed a Gram‐positive reaction, which is characteristic of *S. aureus*. Further, 85% of the Gram‐positive isolates were catalase‐positive, confirming their identity as Staphylococci. Of the catalase‐positive isolates, 92% fermented mannitol, producing yellow zones a typical trait of *S. aureus*. Moreover, 99% of mannitol‐fermenting isolates tested coagulase positive, and all of them (100%) were VP positive, strongly confirming the presence of *S. aureus*) (Table 5).

**TABLE 5:** Biochemical characteristics of isolated *Staphylococcus aureus* (*n* = 301).

Test performed	Positive samples (*n*)	Percentage
Beta haemolysis	120	40
Gram‐positive reaction	102	85
Catalase test	87	85
Mannitol fermentation	80	92
Coagulase test	79	99
Voges–Proskauer test (VP test)	79	100

### 3.6 Questionnaire Survey Results

#### 3.6.1 Demographic Characteristics of the Participants

From a total of 90 respondents 66 fishermen and 24 restaurant workers engaged in fishing activity and fish origin food processers were interviewed in the study area. Out of participants 76 (84.44%) were males and also 58 (64.44%) were literate (Table 6).

**TABLE 6:** Demographic characteristics of the participants (*n* = 90).

Variables	Description	No. of respondents (%)
Sex	Male	76 (84.44)
	Female	14 (15.55)
Age	20–30	26 (28.88)
	31–40	41 (45.55)
	>40	23 (25.55)
Educational status	Literate	58 (64.44)
	Illiterate	32 (35.55)
Years of business‐experience	1–2 years	20 (22.22)
	3–5 years	24 (26.66)
	6–10 years	24 (26.66)
	Above 10 years	22 (24.44)

#### 3.6.2 Questionnaire for Fish Harvesters at Unloading Sites

Total number of the respondents transport fishes without ice by using a plastic bag and most of them harvest Nile tilapia fish 41 (62%). Above 60 (90%) of the respondents did not wash or clean their boats before and after starting of fishing activity and 54 (81.8%) of the respondents had known on improper transportation of fish and improper use of hooks and filleting boards can be a source of fish food contamination. Most of the fishery men were sold the caught fishes within 6–12 h (Table 7).

**TABLE 7:** Questionnaire about food safety for fish harvesters and filters at unloading sites (*n* = 66).

Statements	Value	No. of respondents (%)
Improper transportation, improper use of hooks and filleting boards	Yes	54 (81.8)
	No	12 (18.2)
Transportation of fish to the next chain	With ice	0 (0)
	Without ice	66 (100)
Time to market all harvested fish	6–12 h	38 (57.57)
	2–6 h	28 (42.42)
Type of containers used to carry fish	Plastic bag	16 (24.24)
	Wooden basket	18 (27.27)
	Others	32 (48.48)
Wash hands before and after handling of fish	Yes	45 (68.18)
	No	21 (31.81)
Wash hands after using toilet	Yes	66 (100)
	No	0 (0)

#### 3.6.3 Data Obtained by Direct Observation on Fish Handlers

The observational assessment of hygienic practices among fish handlers in restaurant kitchens (Table 8) provides insight into compliance with basic food safety standards. Out of 24 workers observed, 58.33% (*n* = 14) washed their hands before starting work, whereas 41.66% (*n* = 10) did not. All workers (100%) were free from visible discharge from the nose, eyes, ears or coughing, indicating no obvious signs of illness during handling. The majority, 87.5% (*n* = 21), did not wear jewellery or rings while working, which is a good hygienic practice. Additionally, 79.16% (*n* = 19) wore appropriate hair covers, and 66.66% (*n* = 16) wore proper overcoats. In terms of cleanliness, 75% (*n* = 18) of workers had visibly clean overcoats and body parts. These observations suggest moderate adherence to hygiene practices, though gaps remain in hand washing and the consistent use of protective clothing.

**TABLE 8:** Direct observation on fish handlers in restaurants (*n* = 24).

Observational points	Value	Frequency	Percentage
Washing of hand before starting work	Yes	14	58.33
	No	10	41.66
Discharge from nose, eye, ear and coughing	Observed	0	0
	Not observed	24	100
Wear of jewellery or ring	Observed	21	87.5
	Not observed	3	12.5
Wear of appropriate hair covers	Yes	19	79.16
	No	5	20.83
Wear of appropriate overcoat	Yes	16	66.66
	No	8	33.33
Cleanness of overcoat and visible body part	Clean	18	75
	Not clean	6	25

### 3.7 Antimicrobial Susceptibility Profile

During this study all 79 *S. aureus* isolates were tested against seven antimicrobial agents. The results showed that all isolates (100%) were fully susceptible to ciprofloxacin, indicating its strong effectiveness. Chloramphenicol also demonstrated high effectiveness, with 92% susceptibility, whereas vancomycin and gentamycin showed moderate susceptibility at 84% and 68%, respectively. Conversely, the isolates exhibited the highest resistance to penicillin G, with 87% resistant strains, followed by tetracycline (72% resistance) and erythromycin (55% resistance). These findings suggest that ciprofloxacin and chloramphenicol are the most effective drugs for treating *S. aureus* infections in this context, whereas penicillin G, tetracycline and erythromycin may have limited therapeutic value due to high resistance levels (Table 9).

**TABLE 9:** Susceptibility of *Staphylococcus aureus* isolates against some selected antimicrobials.

Antimicrobial drugs	Antimicrobial concentration	Susceptibility pattern of *Staphylococcus aureus*		
		Susceptible	Intermediate	Resistant
Ciprofloxacin	5 µg	79 (100%)	0 (0%)	0 (0%)
Chloramphenicol	30 µg	73 (92%)	2 (3%)	4 (5%)
Vancomycin	30 µg	66 (84%)	8 (11%)	5 (6%)
Gentamycin	10 µg	54 (68%)	11 (14%)	14 (18%)
Erythromycin	15 µg	22 (28%)	13 (17%)	44 (55%)
Tetracycline	30 µg	14 (17%)	8 (11%)	57 (72%)
Penicillin G	10 U	7 (9%)	3 (4%)	69 (87%)

According to the current investigation 57% (*n* = 45) of *S. aureus* samples tested positive for MDR. The results of the antibiotic susceptibility tests indicate that the isolates exhibited traits of a general pattern of MDR. The highest MDR of drugs which used during susceptibility test (penicillin G, tetracycline and erythromycin).

This study observed that the patterns of antimicrobial resistance among *S. aureus* isolates from fish and fish contact surfaces revealed that MDR is common. The majority of resistant isolates (44%) showed resistance to three antibiotics, with the most frequent combination being penicillin G, tetracycline and erythromycin, found in 37% of isolates. Resistance to four drugs was less frequent, observed in 5% of isolates, involving combinations such as tetracycline, penicillin G, erythromycin and gentamycin or vancomycin. Additionally, resistance to five antibiotics was detected in 8% of the isolates, including chloramphenicol alongside other drugs. These results indicate a significant presence of MDR *S. aureus* strains, which could pose challenges for effective treatment (Table 10).

**TABLE 10:** Patterns of drug resistance of *Staphylococcus aureus* isolated from fish and fish contact surfaces.

Frequencies	Antimicrobial's resistance pattern	No. of resistant	%
Three	P, TET, ER	29	37
	P, TET, VAN	1	1
	P, TET, GEN	3	4
	P, ER, GEN	2	3
Total		35	44
Four	TET, P, ER, GEN	2	3
	P, ER, TET, VAN	1	1
	P, TET, VAN, GEN	1	1
Total		4	5
Five	P, TET, ER, GEN, CHL	3	4
	TET, P ER, GEN, VAN	2	3
	P, TET, ER, GEN, CHL	1	1
Total		6	8

Abbreviations: CHL = chloramphenicol; ER = erythromycin; GEN = gentamycin; P = penicillin G; TET = tetracycline; VAN = vancomycin.

## 4 Discussions

The present study assessed the prevalence, biochemical characteristics and antimicrobial susceptibility of *S. aureus* in fish and associated handling environments from the Lake Tana fish value chain to Gondar city. Out of a total of 301 samples, including 201 fish and 100 hand and knife swabs, 26% were positive for *S. aureus*, indicating significant contamination of fish‐origin foods. This prevalence is similar to the global pooled prevalence of 27.8% in seafood (Kong et al. 2022), suggesting that *S. aureus* contamination of fish is a widespread food safety concern. Compared to local studies, the 26% prevalence is lower than the 33.3% reported by Oumer (2021) in the Lake Tana Bahir Dar district but higher than reports from Brazil (16%) (Abrahim et al. 2011), Japan (15%) (Saito et al. 2010), Libya (5.3%) (Hesham et al. 2019), Iran (5.7%) (Mehdi et al. 2012) and India (9%) (Visnuvinayagam et al. 2015). As Otte and Pica‐Ciamarra (2021) reported variations in prevalence may be attributed to differences in water quality, workers’ hygiene standards, training on fish meat‐borne infections and post‐harvest handling techniques. Inadequate personal hygiene, such as infrequent hand washing and failure to use protective equipment like face masks, likely increases cross‐contamination of fish and handling surfaces.

The study found that 20% of workers’ hands and 32% of knives tested positive for *S. aureus*, highlighting the role of food handlers and utensils as significant sources of contamination. The hand contamination rate is higher than 15.4% reported in Cameroon (Esemu et al. 2022) and 18% in South Korea (Yoon et al. 2008), likely reflecting poor hygiene practices, inadequate sanitation facilities and delayed hand washing after handling fish meat. Knife contamination in this study was lower than the 94% reported in Cameroon (Esemu et al. 2022) but higher than reports from South Korea (0%) (Yoon et al. 2008) and Nigeria (0%) (Iyevhobu et al. 2021). Stainless steel knives, commonly used by fishers, have high biofilm‐forming potential, facilitating bacterial persistence, particularly when knives are shared and inadequately washed. Similarly, 31.8% of workers did not clean equipment after eviscerating fish, and 100% transported fish without ice, further contributing to bacterial proliferation. These observations underscore the importance of basic hygiene, clean utensils and cold chain infrastructure in preventing *S. aureus* contamination.

Among the 201 fish samples, 13% of fresh unfilleted fish and 39% of filleted fish tested positive for *S. aureus*, highlighting filleting as a critical point for contamination. The significant difference between filleted and unfilleted fish (OR = 2.98, *p* = 0.026) demonstrates that direct contact with knives, cutting boards and handlers during processing substantially increases microbial risk. This aligns with previous Ethiopian studies indicating higher bacterial loads in processed fish products compared to intact fish (Haile et al. 2020; Abebe et al. 2016). Among species, African catfish showed the highest prevalence (36%), followed by *Labeobarbus* (27%) and Nile tilapia (24%), although differences were not statistically significant (OR = –1.28, *p* = 0.272). This suggests that contamination is more strongly influenced by post‐harvest handling and environmental hygiene than by intrinsic susceptibility of specific fish species, consistent with prior reports from Lake Tana landing sites (Tessema et al. 2020; Abebe et al. 2016).

Spatial variation across landing sites showed Miterha had the highest prevalence (36%), followed by Gondar city (26%), Gorgora (24%) and Sheha (19%), though differences were not statistically significant (OR = 1.24, *p* = 0.157). However, fish from restaurants (26%, *n* = 26) and unloading sites (26%, *n* = 53) showed a statistically significant difference (OR = 2.2, *p* = 0.024), indicating that additional handling and processing steps in restaurants amplify contamination risk. This emphasizes the importance of a One Health approach, considering both environmental and human factors in controlling *S. aureus* along the fish value chain (Dagnew et al. 2012; Alemayehu et al. 2015).

Biochemical analysis confirmed the identity and pathogenic potential of isolates. Among the 79 positive *S. aureus* samples, 40% exhibited beta‐haemolysis, 85% were Gram‐positive cocci forming grape‐like clusters, 85% were catalase positive, 92% fermented mannitol, 99% were coagulase positive and 100% were VP positive. These results align with standard phenotypic identification protocols and previous Ethiopian studies on foods of animal origin (Quinn et al. 2002; Abebe et al. 2016; Alemayehu et al. 2015). The high prevalence of coagulase‐positive strains indicates strong virulence and the potential to cause foodborne outbreaks. Beta‐haemolytic activity in 40% of isolates further underscores pathogenicity (Asmare et al. 2017).

The antimicrobial susceptibility testing results revealed a concerning yet familiar pattern of bacterial resistance among *S. aureus* isolates. All isolates were susceptible to ciprofloxacin (100%), followed by high susceptibility to chloramphenicol (92%), vancomycin (84%) and gentamicin (68%), indicating that these drugs remain among the few effective options for treating *S. aureus* infections in the study area. This finding agrees with previous Ethiopian studies (Kibrom 2017; Abebe et al. 2016), which reported that fluoroquinolones, such as ciprofloxacin, and phenicol antibiotics, such as chloramphenicol, are still relatively less affected by antimicrobial resistance pressure in the country. The complete effectiveness of ciprofloxacin may be attributed to its broad‐spectrum activity and its comparatively limited misuse in aquaculture and community settings, whereas chloramphenicol's high efficacy could be due to its restricted use in veterinary practice because of toxicity concerns in humans, thereby reducing selective pressure on bacteria. Vancomycin's strong performance (84%) is encouraging, as it is often regarded as the last line of defence against methicillin‐resistant *S. aureus* (MRSA). However, the observed partial resistance (16%) suggests the possible emergence of vancomycin‐intermediate strains (VISA), which requires ongoing surveillance. The moderate susceptibility to gentamicin (68%) indicates that aminoglycosides still play a therapeutic role, although increasing resistance may be driven by plasmid‐mediated modifying enzymes.

In contrast, high resistance rates were observed for penicillin G (87%), tetracycline (72%) and erythromycin (55%), reflecting the widespread and often uncontrolled use of these antibiotics in both human and veterinary medicine. Penicillin resistance in *S. aureus* is primarily attributed to the production of β‐lactamase enzymes that hydrolyse the β‐lactam ring, rendering the antibiotic ineffective. Similarly, tetracycline resistance typically results from efflux pumps or ribosomal protection proteins, mechanisms commonly associated with mobile genetic elements that facilitate horizontal gene transfer. Erythromycin resistance, which is often linked to erm genes causing methylation of the 23S rRNA target site, leads to cross‐resistance to macrolides and lincosamides, thereby reducing the range of effective treatment options.

The finding that 57% of the isolates exhibited MDR defined as resistance to three or more antibiotic classes is particularly alarming. MDR *S. aureus* represents a serious public health and food safety threat, especially when isolated from fish destined for human consumption. The contamination of fish with such resistant bacteria likely reflects environmental pollution through antibiotic residues or effluent discharge from aquaculture operations. The handling or consumption of contaminated fish can promote the horizontal transfer of resistance genes to human pathogens, complicating infection management in both humans and animals. These findings underscore the urgent need for effective antimicrobial stewardship to ensure the rational and controlled use of antibiotics. In Ethiopia, as in many developing nations, antibiotics are often misused in livestock production, aquaculture and human healthcare without proper regulatory oversight. Therefore, enforcing strict regulations on antibiotic use in these sectors is essential to mitigate the emergence and spread of resistant strains.

Given the declining efficacy of commonly used antibiotics, alternative and preventive strategies should be prioritized. These include vaccination to prevent bacterial infections in fish, the use of probiotics and prebiotics to enhance host immunity, the development of phytogenic (plant‐based) antimicrobials with bacteriostatic or bactericidal properties and the strengthening of biosecurity and hygiene measures in aquaculture systems to limit bacterial dissemination. Moreover, continuous surveillance of antimicrobial resistance patterns and molecular characterization of key resistance genes such as mecA (methicillin resistance gene), blaZ (Beta‐lactamase gene), tetK (tetracycline resistance efflux gene) and ermC (erythromycin ribosomal methylase gene) are crucial for monitoring the evolution and spread of resistance determinants within aquatic environments.

Overall, these findings align with the One Health perspective, which emphasizes the interconnectedness of human, animal and environmental health. The detection of MDR *S. aureus* in fish demonstrates that antibiotic misuse in one sector can have far‐reaching implications across ecosystems. Addressing this growing challenge requires coordinated efforts among veterinarians, medical professionals, microbiologists and policymakers to promote responsible antibiotic use, strengthen public health surveillance and implement sustainable disease control strategies that safeguard both animal and human health.

Demographic assessment revealed that 84.4% of respondents were male, reflecting the male‐dominated nature of fishing and fish marketing in Ethiopia (Tefera and Sterk 2010; Assefa et al. 2018). Literacy was relatively high (64.4%), which is associated with better knowledge of hygiene practices; however, actual implementation of safe handling was suboptimal, as observed in hand washing, equipment cleaning and transportation practices. Questionnaire data indicated that most fishermen transported fish without ice, did not clean boats and sold fish within 6–12 h, conditions conducive to microbial proliferation (Abebe et al. 2016; Tessema et al. 2017). Observations in restaurants showed partial compliance with hygiene practices, with 58.3% of workers washing hands before starting work, 79% wearing hair covers and 67% wearing overcoats. Despite these measures, gaps in hygiene and sanitation could contribute to contamination of fish with *S. aureus*.

The study's findings reinforce the importance of integrated interventions targeting the entire fish value chain. High contamination levels on hands, knives and fish indicate that human behaviour, environmental sanitation and post‐harvest handling practices are all critical drivers of *S. aureus* prevalence. Cold chain implementation, routine hygiene training, proper utensil sanitation and surveillance for antimicrobial resistance are essential measures to reduce contamination and protect public health. From a One Health perspective, these interventions should address human, animal and environmental health simultaneously to achieve sustainable improvements in fish safety.

In conclusion, the current study demonstrates that *S. aureus* contamination of fish and fish‐handling environments along the Lake Tana Gondar city value chain is substantial (26%), with higher prevalence in filleted fish and contact surfaces such as knives. Biochemical characterization confirmed the presence of virulent strains, and antimicrobial susceptibility testing revealed high MDR rates, emphasizing the public health implications of contamination. The results highlight the interplay between human hygiene, post‐harvest handling, environmental conditions and antimicrobial resistance in shaping contamination patterns. Comparative analysis with previous studies suggests that differences in prevalence and resistance profiles are largely due to variability in hygiene practices, cold chain infrastructure and antibiotic usage. Effective interventions must therefore combine hygiene education, infrastructural improvements and antimicrobial stewardship to reduce contamination risks and mitigate the spread of MDR *S. aureus* in Ethiopia's fish value chain.

## 5 Conclusion and Recommendation

The study found that over one‐fourth of the samples were positive for *S. aureus*, with high contamination detected on knives and hands that came into contact with fish meat. Significant differences in contamination levels were observed between study sites, particularly at Lake Tana unloading sites and restaurants in Gondar City, as well as between fresh unfilleted and filleted fish samples. The contamination was associated with the use of bare hands, poor hygiene and inadequate sanitation practices. Testing revealed high resistance to penicillin and tetracycline in the *S. aureus* isolates. On the basis of these findings, the study recommends improving awareness among fish meat sellers and workers about safe fish meal preparation, handling and distribution; ensuring cleanliness in fish vendor areas; conducting regular monitoring of antibiotic resistance in *S. aureus*; and using ciprofloxacin and chloramphenicol as the treatments of choice for *S. aureus‐*related diseases in humans and animals. Furthermore, continuous training on hygiene and food safety practices should be provided to fishery societies and restaurant workers, and further research should be undertaken to investigate antimicrobial resistance genes in *S. aureus*.

## Author Contributions

**Misganaw Tezera Ayele, Asnakew Mulaw Berihun and Mengesha Ayehu Getnet** collected the sample in the field, processed the laboratory work and wrote the draft of the paper. **Ambaye Kenubih Worku** supervised the paper, and they have commented on the work of the laboratory, organized data, the analysis and the reviewed manuscript. All authors checked and approved the final work of the manuscript. Therefore, all authors have participated equally.

## Funding

The authors have nothing to report.

## Ethics Statement

The current study has been approved for its ethical soundness for the time from November 2023 to March 2024 by the Institutional Ethical Review Board (IRB) of the College of Veterinary Medicine and Animal Sciences, University of Gondar, Ethiopia. And it has been given at reference (Reference No: CVMAS.Sc.16.282026).

## Animal Ethics

This study considers the ethics of the study animal (fish) and the informed consent obtained from study participants was **verbal**. After it has ensured the necessity of this study via communicating with the professors and instructors of the College of Veterinary Medicine and Animal Sciences, University of Gondar, Ethiopia. A short‐term discussion has been held with the local fishermen and the concerned bodies regarding the fish‐catching system without any welfare interference. And the relevance of the study was also known to those local communities. In addition, its contributions regarding ecological health, fish health, water health and finally increasing fish production both in natural aquatics and aquaculture systems through ensuring the health of the water environment and protecting the welfare of fish were ensured by applying proper handling during sample taking.

## Consent

The informed consent obtained from study participants was verbal. All the participants in this research have played a great role in the preparation of this paper, and we have all observed the final product of the finished paper, evaluated any corrections and updates, and finally, assured that the paper is very good. So, at the end of the day, all authors decided, as we have great consent for the publication of this paper. I confirm the corresponding author has read the journal policies and submit this manuscript in accordance with those policies.

## Conflicts of Interest

The authors declare no conflicts of interest.

## Supporting information




**Supporting File 1**: vms370880‐sup‐0001‐SupMat.docx

## Data Availability

The data to this research is available right now on the **excel spread sheet** and we could submit when there will be any request from the journal editorial board. Additionally, all authors are ready to give the available data to the readers by requesting via email and any communication platform.
