# Supplementary material for: Isolation, Antibiogram and Factors Associated With Staphylococcus aureus From Fish at the Landing Site and Selected Restaurants in Central Gondar, Ethiopia: One Health Approach
Source: Vet Med Sci. 2026 Mar 13;12(2):e70880. doi: 10.1002/vms3.70880 (PMC13098174; doi:10.1002/vms3.70880)

# 8. ANNEXES

Annex I: Laboratory Reagents, Media, and Preparation

**Buffered Peptone Water (BPW)**

Buffered Peptone Water is a liquid medium recommended by ISO 6579 for increasing the recovery of injured Salmonella species from food and associated samples before selective enrichment and isolation. According to ISO 21528, Buffered Peptone Water is used to detect or enumerate Enterobacteriaceae within foodstuffs. Used as the diluent, Buffered Peptone Water complies with ISO 6887.

Standard formula of buffered peptone water

**Ingredients** **Gram/litre**

Enzymatic Digest of Casein 10.0

Sodium Chloride 5.0

Disodium Hydrogen Phosphate 3.5

Potassium Dihydrogen Phosphate 1.5

Final pH 7.0 ± 0.2 at 25^0^C

**Preparation**

Dehydrated medium Suspend 20.0 g of the powder in 1 liter of distilled or deionized water. Mix well. Heat to boil shacking frequently until completely dissolved. Sterilize in autoclave at 121^0^C for 15 min.

**Test Procedure**

- Suspend the sample in Buffered Peptone Water to make dilutions as required. For pre-enrichment,
- Add sample to Buffered Peptone Water at a ratio of 1:10 or 1:9 depending on the method being used.
- Incubate at 37 ± 1^0^C for 16-20 hours before transfer to selective enrichment media.
- Turbidity indicates microbial growth.

**Blood Agar- Composition, Principle, Uses, and Preparation**

Blood agar is an enriched nutritious medium that supports the growth of fastidious organisms by supplementing it with blood or as a general medium without the blood

Blood Agar- Composition

No Ingredients Gram/litre

1. Peptone 10.0

2. Tryptose 10.0

3. Sodium chloride 5.0

4. Agar 15.0

5. Final pH 7.3+0.2 25

**Principle of blood agar**

Hemolysis are exotoxins produced by bacteria that lyse red blood cells. The hemolytic reaction can be visualized on blood agar plates observing through the bright transmitted light. On blood agar plates colonies of hemolytic bacteria may be surrounded by a clear, colourless zone where the red blood cells have been lysed and the haemoglobin destroyed to a colourless compound and which is beta hemolysis. Other types of bacteria can reduce hemoglobin to met hemoglobin which produces a greenish zone around the colonies and is called alpha hemolysis. Gamma hemolysis is lacking hemolysis where no change in the medium is observed. Sheep blood agar base with added sheep blood was developed to allow maximum recovery of organisms without interfering with their hemolytic reactions. The sheep blood agar base was formulated to be compatible with sheep blood and give improved hemolytic reactions of organisms. Casein enzyme hydrolyses and yeast extract provide nitrogen, carbon, amino acids, and vitamins. Peptic digest of animal tissue (PDAT) is the nitrogen source. Sodium chloride (NaCl) maintains the osmotic balance. Sheep blood agar base showed considerable improvement and the expected beta-hemolytic reactions

**Uses of Blood Agar**

Blood Agar is a general-purpose enriched medium often used to grow fastidious organisms to differentiate bacteria based on their hemolytic properties (β-hemolysis, α-hemolysis and γ-hemolysis (or non-hemolytic)) Blood agar can be also used for the detection of phosphate-producing Staphylococci by adding phenolphthalein phosphate to the medium.

**Preparation of Blood Agar**

Suspend 40 grams of the dehydrated medium in 1000 ml of purified/distilled water. Heat to boiling to dissolve the medium completely. Sterilize by autoclaving at 15 lbs. pressure (121^0^C) for 15 minutes. Cool to 45-50^0^C. Mix well before pouring into sterile Petri plates.

**Result Interpretation on Blood Agar**

The basal medium appears light amber-coloured which might look clear to slightly opalescent gel. After the addition of 5%, v/v sterile defibrinated blood; however, the cherry red-coloured opaque gel is formed on the Petri plates

**Limitation on Blood agar**

The growth of Haemophilus hemolytic is inhibited on Blood agar due to the presence of different inhibitors which can be deactivated only by heating the medium after the addition of the blood. The pattern of hemolysis might differ with the type of blood used. The addition of rabbit or horse blood to the basal medium supports the growth of Haemophilus hemolytic, but the growth resembles that of Streptococcus species and thus should be confirmed.

**Mannitol Salt Agar- Composition, Principle, and Preparation**

Mannitol Salt Agar (MSA) is used as a selective and differential medium for the isolation and identification of *S. aureus* from clinical and non-clinical specimens. It encourages the growth of a group of certain bacteria while inhibiting the growth of others. It is a selective medium prepared according to the recommendations of Chapman for the isolation of presumptive pathogenic staphylococci

The medium will select organisms that can live in areas with a high concentration of salt (sodium chloride) and the fermentation of mannitol, demonstrated by the yellow turn of the pH indicator (phenol red), makes it possible to guide the diagnosis.

Composition of Mannitol Salt Agar

No Ingredients Gms/Liter

1 Sodium Chloride…………………… …..75.0

2 D-Mannitol………………………… ………10.0

3 Beef Extract … ……1.0gm

4 Phenol Red …… ..0.0025gm

5 Agar … …….15.ogm

6 Pancreatic Digest of Casein …………5.0gm

7 Peptic Digest of Animal Tissue………….5.0gm

pH 7.4 ± 0.2 at 25^0^C

Total 111.0025gm

Mannitol Salt Agar contains peptones and beef extract, which supply nitrogen, vitamins, minerals, and amino acids essential for growth. The 7.5% concentration of sodium chloride results in the partial or complete inhibition of bacterial organisms other than staphylococci. Sodium chloride also supplies essential electrolytes for transport and osmotic balance. Mannitol is a fermentable carbohydrate, the fermentation of which leads to acid production, detected by the phenol red indicator, which aids in the differentiation of staphylococcal species. Coagulase-positive staphylococci (e.g., *Staphylococcus aureus*) produce yellow colonies and a surrounding yellow medium while coagulase-negative staphylococci produce red colonies and no colour change of the phenol red indicator. Agar is the solidifying agent.

The addition of 5% v/v Egg Yolk Emulsion enables the detection of lipase activity of staphylococci along with mannitol fermentation. The salt clears the egg yolk emulsion and lipase production is detected as a yellow opaque zone around the colonies.

**Preparation and Method of Use of Mannitol salt Agar**

Suspend 111.02 grams in 1000 ml distilled water. Mix until the suspension is uniform. Heat to boiling to dissolve the medium completely. Sterilize by autoclaving at 15 lbs. pressure (121^0^C) for 15 minutes. Avoid overheating. Cool to 45-50^0^C and shake the medium to oxidize the methylene blue (i.e., to restore its blue colour) and to suspend the flocculent precipitate. Pour into sterile Petri plates. Allow plates to warm to room temperature. The agar surface should be dry before inoculating. Inoculate and streak the specimen as soon as possible after collection. If the specimen to be cultured is on a swab, roll the swab over a small area of the agar surface and streak for isolation with a sterile loop. Incubate plates aerobically at 35-37^0^C for 18-24 hours and protect them from light. Examine plates for colonial morphology. If negative after 24 hours, reincubate an additional 24 hours.

Result Interpretation on Mannitol salt Agar

| **Organism** **Growth results** |
| --- |
|  |
| *Staphylococcus aureus* Yellow colonies; may have a  yellow halo around them colonies  *Staphylococcus epidermidis* Colourless or Red colonies with  Zones  Streptococci Micrococci No growth to trace the growth  Large white to orange  Gram-negative bacteria No growth |

Image of S. aureus Cultivate on mannitol Salt agar. Green, Yellow colony's color


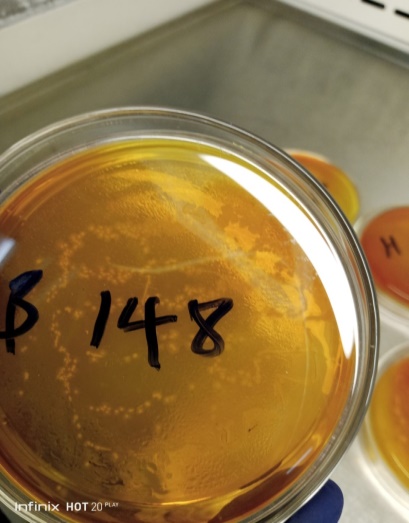

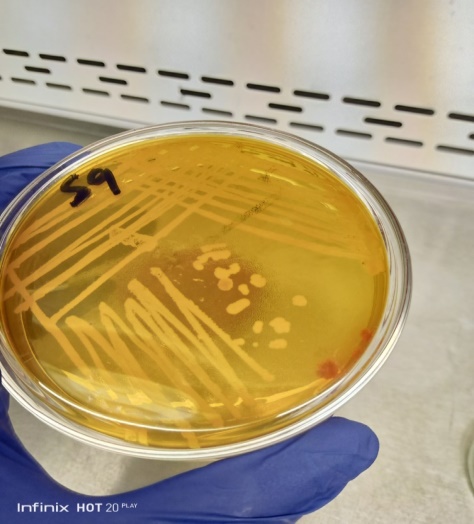


**Uses of Mannitol Salt Agar**

It is used for the selective isolation and differentiation of Staphylococcus aureus from clinical samples. It is also used for the enumeration of staphylococci in food and dairy products. This medium is also included in the Bacteriological Analytical Manual for cosmetics testing and it is also used in the bacteriological examination of swimming pool water, spas and drinking water using membrane filtration.

**Limitations of Mannitol Salt Agar**

Several Staphylococcus species other than aureus are mannitol positive and produce yellow colonies surrounded by yellow zones on this medium (e.g., *S. capitis*, *S. xylosus*, *S. Scohnii*, *S. sciuri*, *S*. *simulans* and other species). Therefore, further biochemical tests are necessary for the identification of *S. aureus* or other species. Most organisms other than staphylococci are inhibited by the high salt concentration found in mannitol salt agar except for some halophilic marine organisms. Few strains of *S. aureus* may exhibit delayed fermentation of mannitol. Negative plates should be re-incubated overnight before discarding. Presumptive *S. aureus* must be confirmed with a coagulase test.

Annex II: Biochemical tests used to isolation of *S. aureus*

**Catalase Test**

Catalase is an enzyme produced by microorganisms that live in oxygenated environments to neutralize toxic forms of oxygen metabolites such as hydrogen peroxide (H2O2). The catalase enzyme neutralizes the bactericidal effects of hydrogen peroxide and protects aerobes or facultative anaerobes. Anaerobes generally lack the catalase enzyme.

**Principle of Catalase Test**

A single colony from a pure culture plate was picked using a sterile loop and mixed with 3% H2O2 on a clean glass slide. Liberation of oxygen in the form of bubbles within a few seconds was taken as a positive test (Das and Mazumder, 2016).

**The procedure of the Catalase test**

1. Transfer a small amount of bacterial colony to a surface of clean, dry glass slide using a loop or sterile wooden stick.

2. Place a drop of 3% H2O2 on to the slide and mix.

3. A positive result is the rapid evolution of oxygen (within 5-10 sec.) as evidenced by bubbling

4. A negative result is no bubbles.

Image of S. aureus Catalase test positive


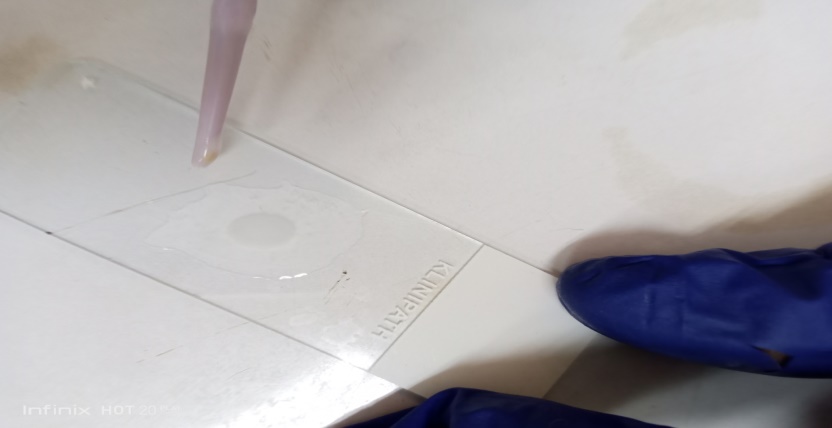


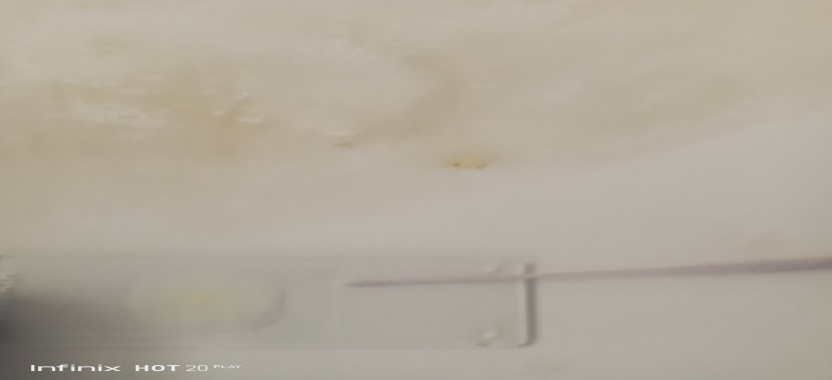


**Gram’s Stain**

The most useful and frequently employed stain in bacteriology is Gram”s staine. Bacteria stained by Gram‟s method fall into Gram positive and Gram negative groups. Gram positive bacteria retain the crystal violet colour and hence appear deep violet or blue in colour. Gram negative bacteria lose crystal violet colour when treated with a decolourizer and are counter stained by safranin and here appear red in colour. There are many bacteria which are Gram variable rather the true Gram positive or Gram negative. The old cultures of some Gram positive organisms are easily decolourized and therefore take the red cooler of counter stain. The Gram positive and Gram negative bacteria differ greatly.

**Procedure**

A) Fix the smear

B) Stain with the primary stain – crystal violet - for 30 seconds

C) Wash crystal violet off with water

D) Add iodine for 10 seconds

E) Wash iodine off with water

F) Decolorize with ethyl alcohol for 10 – 20 seconds (decolorizing agent)

G) Wash the alcohol off with water

H) Counter stain with safranin for 30 seconds

I) Wash the safranin off with water

J) Blot the smear dry

K) Observe under oil immersion

L) When finished, dispose of slides into discard jar

**Objective** • to make certain bacterial" strain” is truly gram positive or gram negative based on the thickness of the cell wall. It divides bacteria into two groups.

**Coagulase Test**

**Tube Coagulase Test Procedure**

Prepare a 1-in-6 dilution of the plasma in saline (0.85% NaCl) and place 1 ml volume of the diluted plasma in small tubes. Emulsify several isolated colonies of test organism in 1 ml of diluted rabbit plasma to give a milky suspension. Incubate the tube at 35^0^C in ambient air for 4 hours. Examine after four hours for clot formation by tilting the tube through 90^0^. By leaving negative tubes at room temperature overnight and re-examining. On positive tubes, plasma is converted into a stiff gel that remains in place when the tube is tilted or inverted and also seen clots are seen floating in the fluid.

Image 7 Tube Coagulase Test on Rabbit plasma


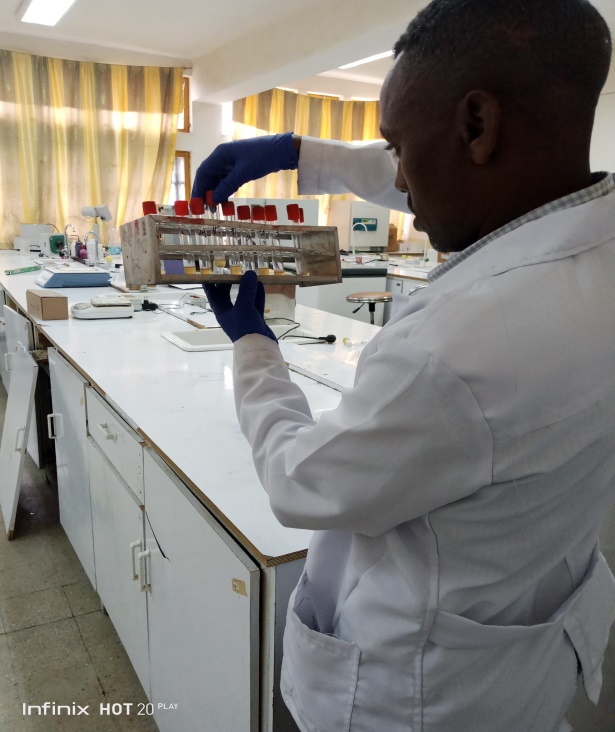

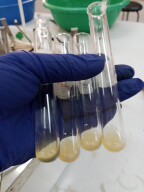


**Slide Coagulase Test**

The organisms were grown on nutrient agar and cultured overnight to obtain pure isolates. One drop of rabbit plasma was spread onto the slide labeled with sample number. The one drop of rabbit plasma was emulsified with the colony (test organisms) using a wire loop then the slide was rocked gently for about ten minutes. Macroscopic clumping was observed in positive suspects while for negatives there were no clumping observed (Harley and Prescott, 2002).

1. Divide the slide into two sections with pencil and should be labeled as test and control.

2. Place a small drop of distilled water on each area.

3. Emulsify one or two colonies of Staphylococcus

4. The test suspension is treated with a drop of citrated plasma and mixed well with a needle.

5. Do not put anything in the other drop that serves as control. The control suspension serves to rule out false positivity due to auto agglutination.

6. Clumping of cocci within 5-10 seconds is taken as positive

7. Positive result is indicated by gelling of the plasma, which remains in place even after inverting the slide.

8. If the test remains negative until four hours at 37^0^C, the tube is kept at room temperature for overnight incubation.

. Image of S. aureus Slide Coagulase Test on Rabbit Plasma


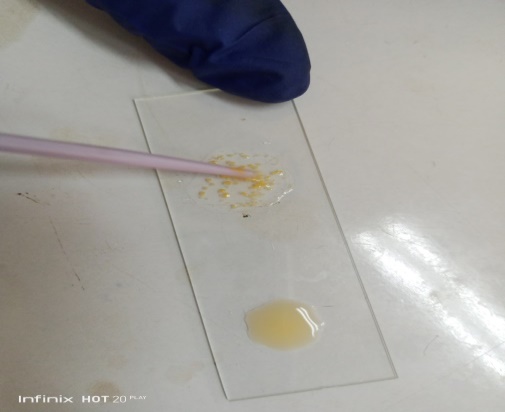

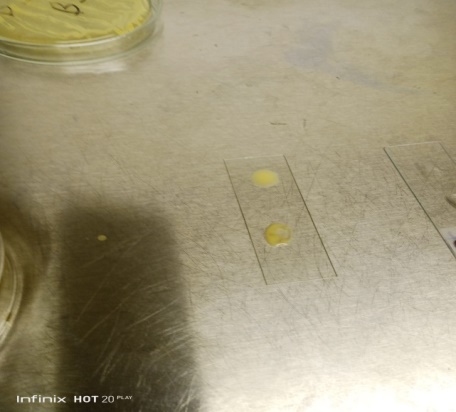


**Vogues Proskauer Test**

**VP test is a biochemical test that detects the ability of bacteria to metabolize the pyruvate into a neutral intermediate product called acetylmethylcarbinol or acetoin.**

**Nutrient Agar**

Nutrient Agar is a general-purpose, nutrient medium used for the cultivation of microbes supporting the growth of a wide range of non-fastidious organisms. Nutrient agar is popular because it can grow a variety of types of bacteria and fungi, and contains many nutrients needed for bacterial growth.

It is an enzymatic digest of animal protein. Peptone is the principal source of organic nitrogen for growing bacteria. The presence of sodium chloride in nutrient agar maintains a salt concentration in the medium that is similar to the cytoplasm of the microorganisms.

Distilled water is essential for the growth and reproduction of micro-organisms and also provides the medium through which various nutrients can be transported. PH is adjusted to neutral (7.4) at 25^0^C.

**Preparation of Nutrient Agar**

Suspend. 28 g of nutrient agar powder in 1 liter of distilled water. Heat this mixture while stirring to fully dissolve all components. Autoclave the dissolved mixture at 121^0^C for 15 minutes. Once the nutrient agar has been autoclaved, allow it to cool but not solidify. Pour nutrient agar into each plate and leave plates on the sterile surface until the agar has solidified. Replace the lid of each Petri dish and store the plates in a refrigerator.

**Uses of Nutrients Agar**

It is frequently used for the isolation and purification of cultures. It can also be used as a means for producing the bacterial lawns needed for antibiotic sensitivity tests. In actuality, antibiotic sensitivity testing is typically performed on media specially formulated for that purpose.

Image of *S. aureus* +cultivate on Nutrients Agar


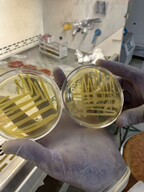


Annex III: Antimicrobial susiptibility test of S. aureus

**Muller Hinton Agar**

Mueller-Hinton agar is considered to be the best for routine susceptibility testing of nonfastidious bacteria for the following reasons:

* It shows acceptable batch-to-batch reproducibility for susceptibility testing.

* It is low for money drugs inhibitors.

* It gives satisfactory growth of most nonfastidious pathogens.

* A large body of data and experience has been collected concerning susceptibility tests performed with this medium.

**Preparation of Mueller-Hinton Agar**

Mueller-Hinton agar preparation includes the following steps.

1. Mueller-Hinton agar should be prepared from a commercially available dehydrated base according to the manufacturer's instructions.

2. Immediately after autoclaving, allow it to cool in a 45 to 50^0^C water bath.

3. Pour the freshly prepared and cooled medium into sterilizes petri dishes on a level, horizontal surface to give a uniform depth of approximately 4 mm. This corresponds to 60 to 70 ml of medium for plates with diameters of 150 mm and 25 to 30 ml for plates with a diameter of 100 mm.

4. The agar medium should be allowed to cool to room temperature and, unless the plate is used the same day, stored in a refrigerator (2 to 8^0^C).

5. Plates should be used within seven days after preparation unless adequate precautions, such as wrapping in plastic, have been taken to minimize drying of the agar.

6. A representative sample of each batch of plates should be examined for sterility by incubating at 30 to 35^0^C for 24 hours or longer.

**Procedures for antimicrobial susceptibility testing of *s. aureus***

- At least 4-5 well isolated colonies of the same morphological type will be selected from the agar plate.
- Just the top of the colonies are touched and the growth transferred to a tube containing saline or tryptone soya broths suspension.
- The turbidity of suspension is adjusted by comparison with a 0.5 McFarland turbidity standard.
- The standard and the test suspension are placed in similar 4-6 ml thin, glass tube or vials.
- The turbidity of the test suspension is adjusted with broth or saline and compared with the turbidity standard, against a white background with contrasting black lines, until the turbidity of the test suspension equates to that of the turbidity standard.
- A sterile, non-toxic swab on an applicator stick is dipped into the standardized suspension of bacteria and excess fluid is expressed by pressing and rotating the swab firmly against the inside of the tube above the fluid level.
- The swab is streaked in three directions and continuously brushed over the Mueller Hinton agar Note:-The surface of the agar should be moist but no droplets of moisture.
- The inoculated plates are allowed to stand for 3-5 minutes, but no longer than 5minutes and the discs are placed onto the agar surface using sterile forceps or an antibiotic disc dispenser.
- Each disc is gently pressed with the point of a sterile forceps to ensure complete contact with the agar surface.
- The discs should be placed no closer together than 24mm (center to center).
- After incubation, the diameters of the zones of inhibition are measured to the nearest mm using a ruler or calipers.
- The diameters are read from the back of the plate and the zones should be read across the center of the discs.

Annex IV: Antimicrobial agents used, disc content and zone of inhibition for interpretive standards for *S. aureus*

| **Antibiotics discs Disc**  **content** | **Zone of inhibition by mm** |
| --- | --- |
|  | **Susceptible Intermediate Resistant** |
| Ciprofloxacin (CIP) 5 μg ≥21 16-20 ≤15  Chloramphenicol 30 μg ≥18 13-17 ≤12  Gentamycin (GEN) 10 μg ≥15 13-14 ≤12  Erythromycin 15μg ≥23 14-22 ≤13  Tetracycline 30μg ≥19 15-18 ≤14  Vancomycin 30μg ≥15 10-14 ≤ 9  Penicillin G 10 Unit ≥29 21-28 ≤ 20 | |

**McFarland Standards**

**Purpose of McFarland Standards**

Turbidity standards called McFarland Standards are used to estimate the number of bacteria in a liquid suspension. The turbidity of a bacterial suspension is visually compared to the turbidity of the relevant standard using the standards. To manufacture standards, sulfuric acid is mixed with barium chloride to create a precipitate of barium. The two reagents' quantities are changed to create standards with varying turbidities, which correspond to varying bacterial concentrations.

**Reagents:**

1. Sulfuric acid, 1%
2. Barium Chloride, 1.175%

**Procedure for the Preparation of a 0.5 McFarland Standard:**

1. Add approximately 85 ml of 1% sulfuric acid (H_2_SO_4_) to a 100ml volumetric flask.
2. Using a volumetric pipette, add 0.5ml of 1.175% anhydrous barium chloride (BaCl_2_) dropwise to the 1% sulfuric acid (H_2_SO_4_) while constantly swirling the flask.
3. Bring the volume to 100ml with 1% H_2_SO_4_.
4. Stir or mix for approximately 3 to 5 minutes while examining visually, until the solution appears homogeneous and free of clumps. A magnetic stirrer can be used for this step if available.
5. Check optical density, following the procedure described in the QC section below and record on QC sheet.
6. If QC is acceptable, dispense 2 to 7 ml volumes (depending on volumes routinely used in test) into each glass screw- cap tube.
7. Label the tubes appropriately including the expiration date and the initials of the person preparing the standards. Make sure that the labeling is positioned so that it does not interfere with spectrophotometer readings.
8. Cap the tubes tightly.
9. Draw a line to mark the meniscus on each tube. This mark can be used as a guide to check for evaporation at a later time.
10. Seal the tubes with paraffin or Parafilm.

**Photos during the research**


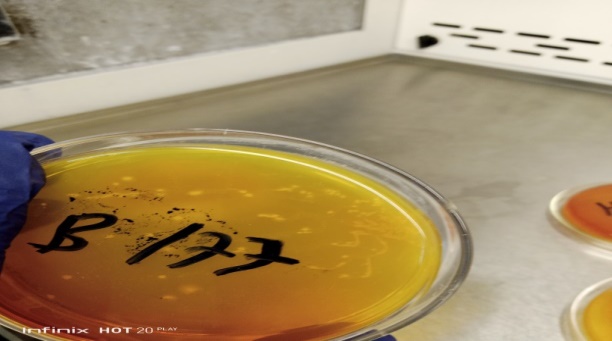
 **
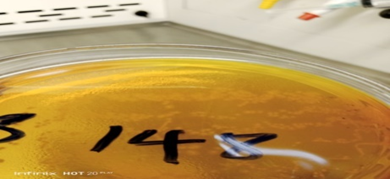
**


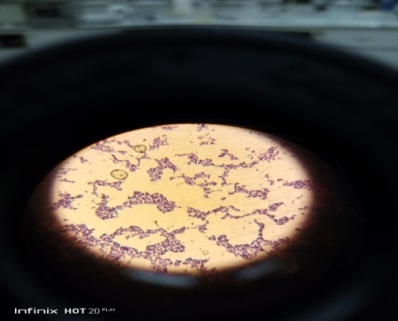

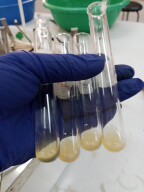


Annex V: Observational points on restaurants during visit and their workers hygienic status and safety

1. Wear of appropriate overcoat. A. Yes B. No

2. Wear of appropriate hair cover. A. Yes B. No

3. Cleanness of overcoat and visible body part.

A. Clean B. Not clean

4. Wear of jewelry or ring. A. Observed B. Not observed

5. Washing of hand before starting work. A. Yes B. No

6. Discharge from nose, eye, ear and coughing.

A. Observed B. Not observed

Annex VI: Survey questions and questionnaire format in hotels and restaurants

Date: _____________ Location: ___________ Respondent’s Code: ___________

1. Sex A. Male B. Female

2. Age A. 20 -30 B. 31-40 C. >41

3. Educational Status A. Illiterate B. Literate,

4. If the answer is “B”, state the level________________

5. Role of respondents A. Owner B. Worker

6. Years of business experience A. 1-2 years B. 3-5 years C. 6-10 years D. above 10years

7. Do you believe people with gastroenteritis and skin abrasion allowed in handling/cooking of fish? A. Yes B. No

8. Do you wash your hands before and after handling of fish? A. Yes B. No

9. Using of clean cutting and filleting board and fly repellant to working area can reduce risk of fish contamination? A. Yes B. No

10. Do you wash hands after using toilet? A. Yes B. No

11. Have you heard about food born disease? A. Yes B. No

12. If yes, what is the cause of the disease? __________________

13. Use of sufficient heat and spice for asalebileb, asadulet…etc. can prevent fish contamination during cooking? A. Yes B. No

14. Covering of hair is important in reducing risk of food contamination during work? A. Yes B. No

15. Do you clean contact surfaces before you start business? A. Yes B. No

16. Using gloves is important in reducing risk of food contamination? A. Yes B. No

17. Using apron is important in reducing risk of food contamination?

A. Yes B. No

18. Raw and cooked foods should be stored separately to reduce risk of food contamination?

A. Yes B. No

19. Do you get food hygiene training for sanitary handling of food? A. Yes B. No

Annex VII: Questionnaire and background information in fishing activity and fish handling at the unloading sites.

Date: __________________Location: ________________ Respondent’s Code: ____________

1. Sex A. Male B. Female

2. Age A. 20 -30 B. 31-40 C. >41

3. Educational Status A. Illiterate B. Literate

4. If the answer is “B”, state the level________________

5. How long have you been in this business?

A. 1-2 years B. 3-5 years C. 6-10 years D. above 10 years

6. Do you think improper transportation of fish, hooks and filleting boards can be a source of food contamination? A. Yes B. No

7. What types of fishes do you harvest more?

A. African Cat fish

B. Labeobarbus species

C Nile tilapia

D. Other specify

8. How long does it take you to transport caught fish from point of fishing to landing?

A. Less than 2 hours

B. More than 6 hours but up to 12 hours

C. 2 hours to 6 hours

9. How do you transport the fish to next chain to wholesaler, retailer or direct to the market?

A. On ice C. In a refrigerated van

B. Without ice D. other specifies.

10. How long after landing you able to market all your catches?

A. Less than 2 hours C. 6 to 12 hours

B. 2 hours to 6 hours D. 12 to 24 hours E. More than 24 hours

11. What containers do you use to carry the raw fish during transportation?

A. wooden Basket B. Ice chests C. plastic bag D. others

12. Do you wash your hands before and after handling of fish?

A. Yes B. No

13. Do you wash hands after using toilet? A. Yes B. No

14. Have you heard about food born disease? A. Yes B. No

15. If yes, what is the cause of the disease? ____________

16. Have you heard about S. aureus as food-borne pathogens?

A. Yes B. No

17. If ‘yes’ what is the symptom it show more frequently___________??

Annex VIII: Ethical Clearance


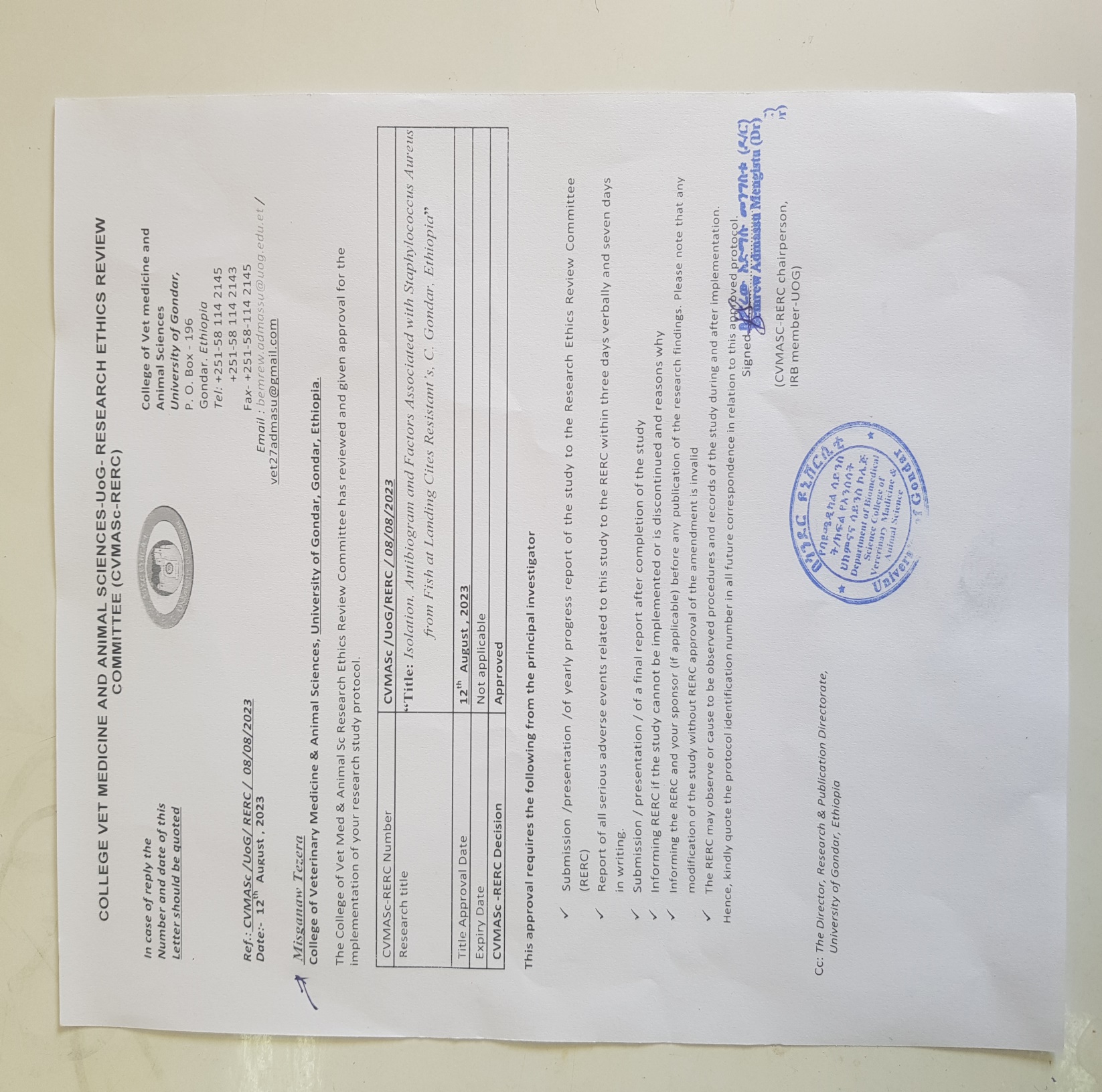

Supplement: Supplementary file 1 — Supporting File 1: vms370880‐sup‐0001‐SupMat.docx [file VMS3-12-e70880-s001.docx]
